# Normal growth of infants receiving an infant formula containing *Lactobacillus reuteri*, galacto-oligosaccharides, and fructo-oligosaccharide: a randomized controlled trial

**DOI:** 10.1186/s40748-015-0008-3

**Published:** 2015-04-07

**Authors:** Le Ye Lee, Roshan Bharani, Agnihotri Biswas, Jiun Lee, Liên-Anh Tran, Sophie Pecquet, Philippe Steenhout

**Affiliations:** Department of Neonatology, National University Health System, NUHS Tower Block 1E Kent Ridge Road, Singapore, 119228 Singapore; Department of Pediatrics, Yong Loo Lin School of Medicine, National University of Singapore, Singapore, Singapore; Nestle Clinical Development Unit, Nestec Ltd, Vevey, Switzerland

**Keywords:** Infant formula, Probiotic, *Lactobacillus reuteri*, Galacto-oligosaccharides, Fructo-oligosaccharide, Growth, Safety

## Abstract

**Background:**

The safety of an infant formula containing a new mixture of the prebiotics galacto-oligosaccharides (GOS) and fructo-oligosaccharide (FOS) and the probiotic *Lactobacillus reuteri* needs to be evaluated.

**Methods:**

Healthy term infants in Singapore were randomly assigned (using computer-generated allocation sequences) to receive exclusively an experimental infant formula containing *L. reuteri*, GOS (5.50 g/L), and FOS (0.36 g/L) or a control formula containing only *L. reuteri* from enrollment (7–14 days of age) to 4 months of age. The primary objective of this trial was to demonstrate that weight change between birth and 4 months of age in infants fed the experimental formula was not inferior to World Health Organization (WHO) Child Growth standards. The non-inferiority margin was −0.5 standard deviations (SD). The secondary objectives were to compare changes in anthropometric measurements (weight, length, body mass index, and head circumference), digestive tolerance, stool bacterial counts, urinary D- and L- lactate concentrations, and adverse events in the two formula groups.

**Results:**

The intention-to-treat (ITT) population included all randomized infants stratified by gender, (experimental group, N = 68 and control group, N = 72). The per-protocol (PP) population included 61 infants in the experimental and 62 infants in the control groups. The change in weight-for-age z-score between birth and 4 months was +0.93 (95% confidence interval [CI]: +0.63 to +1.23) SD in the experimental group and +0.92 (95% CI: +0.62 to +1.22) SD in the control group in the PP population, indicating non-inferior weight gain in both formulas groups compared with WHO standards. The ITT population had similar results. Liquid stools occurred more frequently in the experimental compared with the control group and median bifidobacteria, lactobacilli, and enterococci counts were higher in the experimental group (p < 0.05). Other secondary outcomes were not significantly different between groups.

**Conclusions:**

Infant formula containing *L. reuteri* + GOS/FOS supports normal growth and is safe.

**Trial registration:**

ClinicalTrial.gov: NCT01010113

## Background

Human milk is a superior source of nutrition for infants. It contains nutrients and other bioactive components that have beneficial health effects, which are manifested partly by the reduced susceptibility of breast-fed infants to infections [1-4].

Understanding and replicating some of the beneficial properties of human milk is an important goal in infant formula development. However, progress in this area has been incremental due to the complexity of human milk, our limited knowledge of its bioactive components, and technical challenges in reproducing some of its properties. Nonetheless, it is known that part of the benefits of human milk derive from its ability to stimulate the development of a gut microbiota in infants rich in bifidobacteria and lactobacilli. The initial inocula for infants’ microbiota occurs via the passive transmission of these bacteria from the birth canal and through human milk [5-7]. Human milk also contains a large array of undigestible oligosaccharides, which can selectively stimulate the growth of bifidobacteria and lactobacilli in the infant gut [7,8]. In addition to their bifidogenic properties, the non-digestible oligosaccharides in human milk have other beneficial health effects; for example, some of the sialic acid-containing oligosaccharides have similar structures to glycans on receptors in the gut epithelium that can bind pathogens. Thus, these human milk oligosaccharides bind directly to pathogens and act as decoys thereby, reducing infections [1,9].

Addition of probiotic *Bifidobacterium* or *Lactobacillus* strains as well as non-digestible oligosaccharides (prebiotics) to infant formulas allows at least some mimicking of human milk. Various bacterial strains have been used in the development of probiotc-containing infant formulas. *Lactobacillus reuteri*, a probiotic whose safety and tolerance in both term and preterm infants has been demonstrated in clinical studies [10-14] may also have potential beneficial effects in the management of colic [15-17], in preventing infections, and in reducing diarrhea [18-21].

The prebiotics trans galacto-oligosaccharides (GOS) and fructo-oligosaccharide (FOS) are safe and have previously been shown to increase bifidobacteria counts and decrease counts of harmful pathogens like *Escherichia coli* and *Clostridium* in the gut of infants [22,23]. Furthermore, a prebiotic mixture (8 g/L) containing 90% GOS and 10% FOS was reported to reduce infections in infants during the first 6 months of life [24]. The fermentation products of prebiotics, mainly acetate, butyrate, and propionate, are thought to have immuno-modulatory effects that could, at least in theory, lead to better protection against infections [25].

The mixture of probiotics and prebiotics, synbiotics, has potential synergistic effects [26], which can be used to improve the functional properties of infant formulas. The current study was performed to evaluate the safety of a formula containing *L. reuteri* and GOS and FOS in healthy term infants.

## Population and methods

### Study design

This was a single center, prospective, parallel-group, double-blind, randomized, controlled, non-inferiority trial conducted at the Department of Neonatology of the National University Hospital, Singapore. It took place between November 2009 and June 2011. The trial was performed in accordance with the Helsinki Declaration and complied with Good Clinical Practices as laid out in the International Conference on Harmonization guidelines. It was approved by the institution’s Ethics Committee (the National Healthcare Group, Domain Specific Review Board), and parents/legal guardians signed informed consent forms before infants were enrolled in the study.

The primary objective of this trial was to demonstrate that weight gain between birth and 4 months in healthy term infants fed a formula containing either *L. reuteri* and GOS/FOS or *L. reuteri* only was not inferior to the World Health Organization (WHO) child growth standards [27].

The secondary objective was to demonstrate that daily weight gain in infants fed the formula containing experimental formula with *L. reuteri* and GOS/FOS was not inferior to that of infants fed the control formula containing *L. reuteri* using the non-inferiority margin of −3.0 g/day, the criterion recommended by the American Academy of Pediatrics (AAP) [28]. Other secondary objectives were to compare length, head circumference, and body mass index (BMI) between the two formula groups, assess digestive tolerance, quantify stool bacterial counts and urinary D- and L- lactate concentrations, and assess adverse events (AEs).

### Study population

Newborn infants visiting the National University Hospital of Singapore were recruited for the study if their mothers had elected not to breast feed after hospital discharge. Inclusion criteria further required babies to be healthy, singleton at birth, full term (≥37 weeks and ≤42 weeks of gestation), ≤14 days old, and have a birth weight between 2500 g and 4500 g.

Infants were excluded from the trial if they had any of the following exclusion criteria: congenital illness or malformation that could affect normal growth, significant pre- or post-natal disease, re-hospitalization for >2 days during the first 14 days of life for reasons other than neonatal jaundice, or participation in another clinical trial prior to the beginning of the current one. Additionally, infants whose parents were expected not to be able to comply with the study requirements were also excluded.

### Study formulas and blinding

Formulas were isocaloric (67 kcal/100 ml) and contained bovine milk proteins (40/60 casein/ whey ratio), carbohydrates, namely lactose and maltodextrine, fats (milk fat, vegetable oils from coconut, sunflower, and soya lecithin), vitamins, and minerals in amounts appropriate for full nutrition of newborn infants up to 6 months of age. Both formulas contained *L. reuteri* DSM 17938 at concentrations that would deliver approximately 10^8^ colony forming units (CFU) per day. They differed only in the presence 5.5 g/L of GOS and 0.36 g/L of FOS in the experimental formula without changing the other ingredients. Of note, total available carbohydrates amounts were similar in the two formulas.

Formulas were manufactured by Nestlé in Boué, France. The two formulas were indistinguishable and were supplied in similar cans that were coded with letters and colors by the study sponsor (Nestlé). The infant formula were available in powder form and were provided to the parents during each study visit. Parents, investigator, support staff, and the clinical project manager were blinded to the identity of the formulas.

### Randomization

An in-house computer program (Trial Balance) was used to generate a randomization sequence to allocate infants to the two formula groups. Infants were randomized into the two groups with a 1:1 ratio with stratification by gender. A probability of <0.8 was used to minimize imbalance. The investigator accessed allocation numbers via a web-based application.

### Trial procedure

Recruited infants were randomly assigned to either the control or the experimental group after obtaining informed consent from parent/legal guardian. At baseline (14 days of age) the infants’ birth information (gestational age and mode of delivery), anthropometric measurements (weight, length, and head circumference), and medical history since birth, including any intake of medication, was recorded. Additionally, the parents’ demographic data, weight, and height as well as the mothers’ smoking and drinking habits were also recorded.

At enrollment, parents were given the formulas for their infants along with preparation instructions. Subsequently, the cans were distributed to the patient’s family according to the randomized code. They were also given diaries with explanations on how to record daily volume of formula intake, digestive tolerance, and the occurrence of any illness for the 3 days preceding each visit.

Infants were fed the study formulas *ad libitum* from enrollment to 6 months of age. Visits to the study center took place within 3 days of age 15 days (0.5 month), within 5 days of age 30 days (1 month), within 7 days of age 61 days (2 months), within 7 days of age 122 days (4 months), and within 7 days of age 182 days (6 month) of age. At each visit the study investigator examined the infants, took anthropometric measurements, collected and reviewed the 3-day diaries, and assessed the occurrence of any AEs. Infants with intake of complementary feeding before 4 months and interrupting study formula intake before the 4-month visit were considered to have major protocol deviations. At the 2-month visit, approximately 10 g of fresh stool samples were collected from the first 60 infants enrolled in the study who had had no perinatal antibiotic treatment. The stools were collected at home within 30 minutes after stool emission and transferred into a sterile tube. The container was placed in an aluminium bag as well as a packet of AnaeroGen (Oxoid, Hampshire U.K) to maintain an anaerobic environment. The tightly closed bag will be kept refrigerated (4° Celsius) and transported on ice within 10 hours to the study site. Fresh urine samples were collected during the study visit with the clean catch method.

### Outcome measures

The primary outcome was change in weight between birth weight and 4 months in both the control and experimental arm. All infants were weighed nude on the same electronic scale to the nearest 10 g. The scale was calibrated every month according to the manufacturer’s instructions.

Secondary outcomes were length, BMI, head circumference, digestive tolerance, stool bacterial counts, urinary D- and L-lactate concentrations, and AEs. Recumbent length was measured to the nearest millimeter on a standardized length board with the infants’ feet flexed and with at least two study staff ensuring proper body alignment. Head circumference was measured to the nearest millimeter at approximately 2.5 cm above the eyebrows, at the largest measurement of the head circumference, using a standard plastic-coated, non-stretchable measuring tape.

Digestive tolerance was assessed based on stool frequency and consistency, flatulence, restlessness/irritability, and frequencies of waking up at night and colic.

Total bacterial, bifidobacteria, lactobacilli, *Clostridium*, enteroccci, and *Bacteriodes* counts were quantified by fluorescence in situ hybridization (FISH) using probes described previously [29-37], and *L. reuteri* was quantified by culture plating. For the FISH analysis, 2 g of stool samples were frozen in cryotubes and stored at −80°C until further analysis by an external partner (Biovisible, The Netherlands). For bacterial quantification by culture plating, approximately 2 g of stool was added to a saline solution containing 10% glycerol and frozen immediately in cryotubes at −80°C. Analyses were performed by an external partner (ATT, Piacenza, Italy).

Urine samples were collected at 2 months, and 6-ml aliquots were placed into cryotubes and frozen immediately at −40°C until further analysis. Urinary D- and L-lactate concentrations and creatinine concentrations were determined at the Centre Hospitalier Universitaire Vaudois (Lausanne, Switzerland) as described previously [38,39]. Both D- and L-lactate concentrations were normalized per mole creatinine (mmol lactate/mol creatinine).

### Adverse Events (AEs) and Serious Adverse Events (SAEs)

The study investigator evaluated the seriousness of all AEs and any potential relation to the study products. AEs and SAEs were coded using the WHO Adverse Reactions Terminology (WHO-ART).

### Statistical methods

Sample size was calculated based on the primary outcome of showing non-inferiority in weight gain. The non-inferiority margin was set at −0.5 standard deviation (SD) based on the WHO child growth standards. The type I error (α) was set to 2.5%, power to 80%, and the common SD to 1.0. The difference in mean weight-for-age z-score between groups was expected to be 0.0 SD. Based on a one-sided t-test, 64 infants had to be enrolled in each group to show a significant difference. Assuming a 20% dropout rate, 78 infants had to be enrolled in each group for a total of 156 infants in the study. Sample size was calculated using NQuery Advisor 7.0.

All randomized infants were included in the intention-to-treat (ITT) analysis. Infants with major protocol deviations were excluded from the per protocol (PP) analysis. Data are summarized as mean ± SD or median and interquartile range (IQR).

The primary endpoint was the change in weight-for-age z-scores between birth and 4 months in the two formula groups. Non-inferiority in weight was evaluated based on simultaneous 2-sided 95% CI for the mean weight z-score in each formula group and the CI for the difference in mean change between treatment arms. Changes in weight-for-age z-scores between birth and 4 months were calculated using a mixed model correcting for gender and birth weight and with visit as a random effect. Non-inferiority was established if the lower bound of the one-sided 97.5% confidence interval (CI) lay above −0.5 SD.

The point estimate for the difference between formula groups in mean weight-for-age z-score change from birth to 4 months and the two-sided 95% CI were calculated using a fitted mixed model. The non-inferiority of the experimental formula compared with the *control* formula was tested using −0.5 SD for the lower bound of the 97.5% CI.

Non-inferiority of the experimental formula compared with the *control* formula was also determined by comparing mean daily weight gain in g/day. The non-inferiority margin for the lower bound of the one-sided 97.5% CI for the difference in mean daily weight gain between the formula groups was −3.0 g/day. Changes in other anthropometric measurements (length, BMI, and head circumference) between birth and 4 months were compared between groups using a t-test and a mixed model correcting for gender and birth weight and using visit time (age) as a random effect.

Formula intake, the various parameters for digestive tolerance, and the average number of times of waking up per night were compared between groups using two-sided t-test. Stool counts per day were calculated for each infant by summing the total occurrence of stools and dividing it by the number of days for which it was recorded. To obtain the proportion of a particular stool consistency, the number of days for which a particular consistency was predominant was summed up and divided by the total number of days for which consistency was assessed. Flatulence was considered to have occurred if it occurred ≥2 times per day, irritability if infants cried for ≥3 hours per day, and colic and spitting up if each occurred ≥1 time per day. For each of these symptoms, the proportion of days in which the symptom occurred was the total number of days in which it occurred divided by the total number of days it was assessed.

Mean bacterial counts were compared between groups using a two-sided t-test, with a significance level of 0.05. The percentage of infants having at least one AE or SAE during the 6-month study period was compared between groups using Chi-Square test.

The R statistical package (version 2.13.1) was used in all analyses.

## Results

### Demographics and baseline characteristics

**Figure 1:**
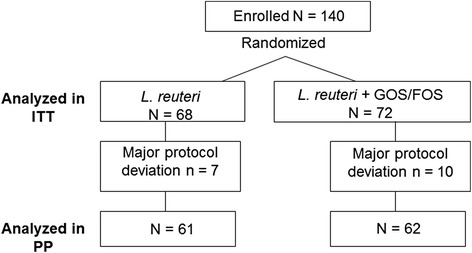
**Infants participating in the study.** ITT, intention to treat; PP, per protocol; GOS, galacto-oligosaccharides; FOS, fructo-oligosaccharide.

One hundred and forty infants were randomized into the two formula groups and were included in the ITT population (Figure 1). A total of 17 infants were excluded from the PP population either because they had intake of complementary food before 4 months or because they interrupted intake of the study formula; therefore 123 infants were included in the PP population (Figure 1).

The demographics and baseline characteristics of infants and their parents were balanced between the two groups, though a higher proportion of households in the experimental group had a smoker (Table 1).

**Table 1:** **Demographics and baseline characteristics**

**Characteristics mean (SD) or number (%)**	***L. reuteri (Control)*** **(N = 68)**	***L. reuteri*** **+ GOS/FOS (Experiment) (N = 72)**
**Age (days) at recruitment**	14.9 (2.1)	14.6 (2.0)
**Male**	28 (41.18)	30 (41.67)
**Gestational Age (weeks) at birth**	38.77 (1.15)	39.19 (1.14)
**Vaginal Delivery**	53 (77.94)	59 (81.94)
**Birth Weight (kg)**	3.13 (0.39)	3.19 (0.41)
**Length at birth (cm)**	49.9 (2.1)	50.3 (1.8)
**Occipital frontal circumference at birth (cm)**	33.3 (1.3)	33.5 (1.3)
**Body Mass Index (kg/m** ^**2**^ **)**	12.53 (1.07)	12.58 (1.13)
**Ethnic Origin: Malay**	47 (69.1)	55 (73.4)
**Asian**	9 (13.2)	8 (11.1)
**Other**	12 (17.6)	9(12.5)
**Households with smoker**	41 (61.2)	49 (70.0)

SD, Standard deviation; GOS, Galacto-oligosaccharides; FOS, Fructo-oligosaccharide.

### Weight gain

Mean weight-for age z-scores between birth and 4 months were above the WHO standards in both formula groups (data not shown). The change in mean weight between birth and 4 months was close to 1 SD in both formula groups (Table 2). The lower bound of the 95% CI of the change in weight-for-age z-scores was above −0.5 SD (relative to WHO standard) in both formula groups in the ITT and PP populations (Table 2).

**Table 2:** **Mean weight gain between birth and 4 months**

	***L. reuteri (control)***	***L. reuteri*** **+ GOS/FOS (experimental)**	**Difference between control and experimental**
**ITT**	**N = 68**	**N = 72**	
**z-score (95% CI)**	+0.82 (0.53 to 1.10)	+0.91 (0.63 to 1.20)	+0.10 (−0.31 to 0.50)
**g/day (SD)**	30.61 (6.5)	31.84 (7.4)	P = 0.302
**PP**	**N = 61**	**N = 62**	
**z-score (95% CI)**	+0.92 (0.62 to 1.22)	+0.93 (0.63 to 1.23)	+0.01 (−0.41 to 0.43)

GOS, Galacto-oligosaccharides; FOS, Fructo-oligosaccharide; ITT, Intention-to-treat; CI, Confidence interval; PP, Per protocol.

Additionally, the lower bound of the 2-sided 95% CI of the difference in the change in mean weight-for-age z-scores between the two formula groups was above −0.5 SD in both the ITT and PP populations (Table 2), demonstrating the non-inferiority of the experimental formula compared with the *control* formula based on the pre-specified margin. Analysis of a mixed model confirmed no significant treatment effect (p = 0.755).

Infants in the experimental group had slightly greater mean weight gain compared with those in the control group (31.8 g/day vs. 30.6 g/day). However, the difference in mean daily weight gain in g/day was not significantly different between the two groups (Table 2).

### Changes in other anthropometric measurements

Changes in length, head circumference, and BMI between birth and 4 months were not significantly different between the two groups; though BMI tended to be higher in the experimental group (Table 3).

**Table 3:** **Change in length, head circumference, and BMI measurements between birth and 4 months, ITT**

	***L. reuteri*** **(control) (N = 67)**	***L. reuteri*** **+ GOS/FOS (Experimental) (N = 70)**	P-value*
**Mean (SD)**			
**Length, mm/day**	+1.2 (0.189)	+1.2 (0.194)	0.824
**Head Circumference, mm/day**	+0.70 (0.114)	+0.72 (0.134)	0.332
**BMI kg/m** ^**2**^	3.69 (1.327)	4.0 (1.940)	0.281

*t-test; BMI, Body mass index; ITT, Intention-to-treat; SD, Standard deviation; GOS, Galacto-oligosaccharides; FOS, Fructo-oligosaccharide.

### Formula intake and digestive tolerance

**Figure 2:**
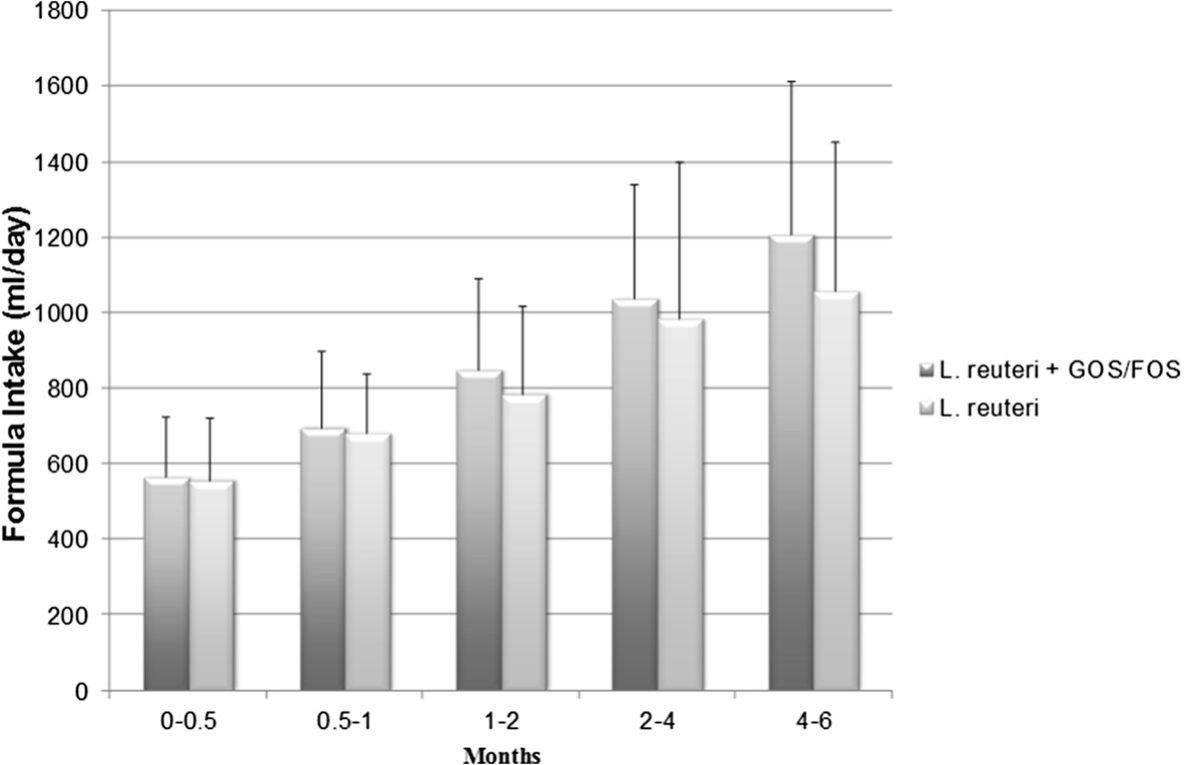
**Mean daily volume of formula intake.** Error bars indicate standard deviation. GOS, galacto-oligosaccharides; FOS, fructo-oligosaccharide.

Mean daily formula intake was not significantly different between the two groups at any time during the study, though overall intake seemed to be greater in the experimental group throughout the study (Figure 2). Other than liquid stools, which occurred more frequently in the experimental group (Table 4), other parameters for digestive tolerance showed no significant difference that would indicate reduced tolerance for the experimental formula (Table 4).

**Table 4:** **Measures of digestive tolerance during the first 4 months, ITT**

	***L. reuteri (control)***		***L. reuteri*** **+ GOS/FOS (experimental)**		P-value*
	**N**	**Mean (SD) daily frequency**	**N**	**Mean (SD) daily frequency**	
**Stool Frequency**	61	4.84 (6.06)	62	6.06 (7.52)	0.323
**Hard stools**	61	0.42 (1.81)	62	0.13 (0.81)	0.256
**Liquid stools**	61	0.11 (0.39)	62	0.43 (0.92)	0.014
**Flatulence**	61	0.91 (0.20)	62	0.93 (0.18)	0.561
**Irritability**	30	0.02 (0.04)	25	0.02 (0.04)	1.000
**Spitting**	42	0.75 (1.14)	43	0.65 (0.74)	0.566
**Colic**	14	0.26 (0.19)	15	0.33 (0.33)	0.152
**Waking up**	61	1.94 (0.74)	62	2.09 (0.59)	0.217

*two-sided t-test; ITT, Intention-to-treat; GOS, Galacto-oligosaccharides; FOS, Fructo-oligosaccharide; SD, Standard deviation.

### Stool bacterial counts

Median bifidobacterial counts were about half a log higher in the experimental group (Table 5), and they made up a larger proportion of the stool bacterial population in the experimental group (83.8%) as compared with the control group (39%). Median counts of other bacteria as well as their proportions of the total bacterial counts were comparable between the two groups (Table 5).

**Table 5:** **Stool bacterial counts (CFU/g) at 2 months**

	***L. reuteri (control)***		***L. reuteri*** **+ GOS/FOS (experimental)**		Comparison of bacterial counts between groups*
	**N (% BDL)** ^**†**^	**Median (IQR)**	**N (% BDL)** ^**†**^	**Median (IQR)**	
**Total bacterial count**	32 (0)	2.4 × 10^10^ (1.7 × 10^10^)	29 (0)	3.7 × 10^10^ (3.2 × 10^10^)	0.01
***Lactobacillus reuteri***	32 (0)	9.5 × 10^6^ (1.5 × 10^7^)	29 (3.45)	1.5 × 10^7^ (4.3 × 10^7^)	0.118
**Bifidobacteria**	32 (0)	9.4 × 10^9^ (1.3 × 10^10^)	28 (3.45)	3.1 × 10^10^ (2.4 × 10^10^)	0.00
**Lactobacilli and Enterobacteria**	28 (12.5)	5.2 × 10^7^ (8.5 × 10^7^)	26 (10.34)	1.0 × 10^8^ (1.9 × 10^8^)	0.07
**Enterobacteria**	32(0)	5.1 × 10^8^ (6.5 × 10^8^)	29 (0)	7.6 × 10^8^ (8.4 × 10^8^)	0.132
**Clostridia**	21 (34.38)	4.8 × 10^8^ (9.3 × 10^8^)	14 (51.72)	2.6 × 10^8^ (1.7 × 10^9^)	0.752

*Two-sided t-test; ^**†**^Number of samples analyzed and proportion below detection limit (BDL); CFU, Colony forming unit; IQR, Interquartile range.

### Urinary D- and L- lactate concentrations

Mean (± SD) D-lactate concentrations in the experimental (4.0 ± 5.4 mmol/mol creatinine) and the control groups (3.1 ± 3.1 mmol/mol creatinine) were not significantly different (t-test, p = 0.46). Similarly, mean (± SD) L-lactate concentrations (58.6 ± 58.0 and 66.2 ± 86.9 mmol/mol creatinine in the experimental and the control groups, respectively) were not significantly different between the two groups (t-test, p = 0.69).

### AEs

Fifty-two infants (76.5%, ITT) in the control group and 53 (73.6%, ITT) in the experimental group had at least one AE during the study (Chi square P > 0.5). The most frequent AEs (by system organ class) in both groups were upper respiratory tract infections (45% of infants), respiratory system disorders (26% of infants), skin and appendages disorders (16% of infants), general disorders (14%), and resistance mechanism disorders (10%). Slightly more infants in the experimental group had gastro-intestinal (GI) system disorders and skin and appendages disorders (8% and 19%, respectively) compared with those in the *control* group (6% and 13%, respectively). More infants in the *control* group (13%) had resistance mechanism disorders compared with those in the experimental (7%). None of these differences were statistically significant.

SAEs (comprising hospitalization for various infections) were reported in five (7.4%) infants in the *L. reuteri* group and 10 (13.9%) infants in the experimental group (Chi Square, P = 0.28). The number of SAE events reported were 7 out of 68 control infants and 14 out of 72 experimental infants were also not significant (Chi Square, P = 0.16) (Table 6). None of the AEs or SAEs was reported to be related to the study formulas.

**Table 6:** Number of Serious Adverse Events (SAEs)* reported during the study, Intention-to-Treat

SAE preferred term**	***L. reuteri (control)*** **(N = 68)**	***L. reuteri*** **+ GOS/FOS (experimental) (N = 72)**
**Total number of SAEs**	7	14
**Infection Viral**	1	0
**Influenza-Like Symptoms**	1	0
**Pneumonia (CXR/bacterial)**	2	3
**Vomiting**	1	0
**Varicella Zoster infection**	1	0
**Choking**	1	0
**Meningitis (aseptic)**	0	1
**Fever (>38.0 degrees Celsius)**	0	4
**Urinary Tract Infection (Bacterial)**	0	1
**Upper Respiratory Tract Infection**	0	1
**Rash Erythematous**	0	1
**Bronchitis**	0	1
**Apnoea Neonatal**	0	1
**Pharyngitis**	0	1

*Some infants had >1 SAE; **Coded according to World Health Organization Adverse Reactions Terminology.

## Discussion

Formulas containing synbiotics are thought to have more beneficial properties than those containing probiotics or prebiotics separately [26]. Even though no safety or tolerance issues with the use of either probiotics or the prebiotics GOS and FOS have been reported in healthy term infants, because of the paucity of studies with synbiotics, the ESPGHAN committee on Nutrition has reiterated the need for well-conducted clinical trials evaluating the safety and efficacy of synbiotic products [40].

*L. reuteri* has been evaluated in clinical trials in infants with no reported safety issues [10,12,26]. Similarly, the safety of the prebiotic GOS/FOS mixture has also been accepted and is authorized for use in infant formulas. At the initiation of our study, the safety of the mix of *L. reuteri* and GOS/FOS for infants had not, to our knowledge, been studied. The aim of the current study was to evaluate the safety of a formula containing *L. reuteri* and GOS and FOS in healthy term infants.

Our study showed that weight gain between birth and 4 months in healthy term infants fed experimental infant formula containing *L. reuteri* + GOS/FOS was not inferior to the WHO standards nor to that of infants fed a control formula containing only *L. reuteri*, indicating the safety of the synbiotic formula with respect to its nutritive value and tolerance data. Consistent with this, neither daily length nor head circumference gains, between birth and 4 months nor changes in BMI between birth and 4 months showed any significant difference between the two formula groups in this study. Volume of formula intake tended to be slightly higher in the experimental group throughout the study. This may have partly contributed to the higher, but statistically non-significant, weight gain in infants fed the experimental formula.

Overall, there were no differences in GI symptoms among infants consuming the two formulas, indicating a good tolerance of the synbiotic formula. The frequency of colic and waking up at night, which could be indications of GI discomfort, were infrequent and similar in both groups. In a study published recently we showed that infants fed a control formula or a formula containing *L. reuteri* woke up approximately twice nightly [14], consistent with the current study. Furthermore, although infants on the synbiotic formula were more likely to have loose stools, daily stool output was similar between both groups. This GI symptom is consistent with the effect of GOS and FOS previously described, and is also commonly seen among breast-fed infants [41-44]. None of the infants in the study required hospital admission for diarrheal illness.

Both the bifidobacterial counts and the relative proportion of bifidobacteria in stool were higher in the experimental group compared with the control group, consistent with the bifidogenic effect of GOS and FOS previously reported [44,45]. Counts of the other bacterial species in the two groups were within half a log of each other.

Although more infants in the experimental group had SAE compared with the control group, these did not appear to be related to the formula, nor were the differences significant. All SAEs were related to short duration hospitalizations and none of the infants died or had long term harm or disability as a result of an SAE. All children had a clinical review post SAE and all had recovered well subsequently. Additionally none of the infants had diarrhea, and none of the SAEs (except a case of vomiting in the *L. reuteri* group) were related to the GI system.

## Conclusions

The synbiotic experimental formula containing *L. reuteri* + GOS/FOS was safe and well-tolerated in healthy term infants. Growth of infants consuming the synbiotic formula was not inferior to WHO growth standards or to that of infants consuming the formula containing only the probiotic.

## Abbreviations

GOS: Galacto-oligosaccharides
FOS: Fructo-oligosaccharide
WHO: World Health Organization
SD: Standard deviations
ITT: Intention-to-treat
PP: Per-protocol
CI: Confidence interval
AAP: American association of pediatrics
BMI: Body mass index
AEs: Adverse events
CFU: Colony forming units
FISH: Fluorescence in situ hybridization
SAEs: Serious adverse events
WHO-ART: WHO adverse reactions terminology
IQR: Interquartile range
GI: Gastro-intestinal
ESPGHAN: European Society of Paediatric Gastroenterology, Hepatology and Nutrition
